# High stocking density triggers stress-induced physiological changes and alters nasal and fecal microbiota in finishing pigs

**DOI:** 10.1186/s40813-026-00515-3

**Published:** 2026-04-24

**Authors:** Robie Vasquez, Van Aldren Cañas, Sungbo Cho, Ji Hoon Song, Jae Seung Lee, Chaibin Lim, Dae-Kyung Kang, In Ho Kim

**Affiliations:** 1 Department of Animal Biotechnology, Dankook University, Cheonan, 31116 Republic of Korea; 2 Smart Animal Bio Institute, Dankook University, Cheonan, 31116 Republic of Korea

**Keywords:** Crowding, Fecal microbiota, Growth, Housing, Metabolites, Nasal microbiota, Pig, Stocking density, Stress

## Abstract

**Background:**

High stocking density in pig production is associated with stress and reduced growth, but its effects on nasal and fecal bacterial communities, gut metabolites, and predicted functions are not fully understood. We hypothesized that high stocking density disrupts host–microbiota interactions, contributing to stress and impaired growth. In this study, 98 finishing pigs [(Landrace × Yorkshire) × Duroc; 21 weeks old] were assigned to high stocking density (HD: 5 pigs per pen, 0.58 m^2^ per pig, *k* = 0.029) or control (CON: 3 pigs per pen, 0.97 m^2^ per pig, *k* = 0.049) in a completely randomized block design. Growth, blood profile, and coughing index were monitored over 4 weeks. Nasal and fecal microbiota were characterized using full-length 16S rRNA sequencing, fecal metabolites were quantified by high-performance liquid chromatography, and correlations with physiological parameters were analyzed.

**Results:**

After 4 weeks, pigs in HD group had lower final body weight (*P* = 0.048), average daily gain (*P* = 0.035), and average daily feed intake (*P* = 0.048), alongside elevated cortisol (*P* = 0.037) and coughing index (*P* < 0.001). Alpha-diversity of the nasal microbiota was reduced (Shannon Entropy, *P* = 0.009), while the fecal microbiota showed non-significant decrease. Beta-diversity revealed distinct clustering between CON and HD in nasal (*P* = 0.003; R^2^ = 0.021) and fecal (*P* = 0.005; R^2^ = 0.042) bacterial communities. Taxonomic profiling of the HD group revealed enrichment of *Rothia nasimurium*, *Psychrobacter faecalis*, and *Globicatella* sp. in nasal microbiota, and increased *Clostridium sensu stricto* 1 and *Terrisporobacter*, with marked decline in *Lactobacillus*, particularly *L. amylovorus* and *L. johnsonii*, in fecal microbiota (*P* < 0.05). Metabolism-related functional pathways were altered in both bacterial communities. Fecal lactate was decreased (*P* = 0.005) and was positively correlated with tumor necrosis factor-α (TNF-α) (*P* = 0.048). Biomarkers in the HD group were negatively correlated with growth performance, whereas those in the CON group showed positive correlations (*P* < 0.05).

**Conclusions:**

High stocking density impaired growth, increased blood cortisol and stress-related coughing, and altered nasal and fecal bacterial communities in finishing pigs, highlighting potential impacts of crowding on pig productivity.

**Supplementary Information:**

The online version contains supplementary material available at 10.1186/s40813-026-00515-3.

## Background

Pigs are sensitive to biological and physical stressors in the environment, which can affect their welfare, immune responses, and growth performance [22, 45]. In intensive production systems, high stocking density is a common management practice aimed at improving pen utilization and economic efficiency. Research on the effects of stocking density on pig dates to the 1980s, when an allometric equation was introduced to describe how an animal’s physical size (static space) and the area required for social interactions and non-locomotor movements (dynamic space) influence growth performance [38, 7]. This equation remains widely used to date and is supported by studies showing that high stocking density is associated with reduced feed intake, slower growth, and altered stress markers, including cortisol and pro-inflammatory cytokines [19, 26]. Behavioral changes such as increased aggression, social conflict, and tail biting, as well as reduced exploratory activity and physical comfort, are also reported under crowded conditions [10, 7]. These studies suggest that high stocking density housing may compromise pig welfare and productivity, but its effects on microbial communities, particularly in the gut and nasal cavity, are less well understood.

The pig microbiota plays a crucial role in host health by supporting nutrient metabolism, immune function, and the production of metabolites that can modulate growth and stress responses [15, 52, 56]. Consequently, stress can influence microbial composition through the gut–brain axis, potentially altering microbial metabolites and host physiology [18, 31]. In pigs, evidence is beginning to show that environmental and management factors such as space allowance and social stress can impact the intestinal bacterial communities. For example, Liet al. [25] and Kim et al. [20] reported that pigs raised under high stocking density exhibited significant shifts in gut microbial community structure, accompanied by impaired intestinal morphology. These findings indicate that high stocking density can reshape the gut microbiota, although the extent and functional consequences of these changes remain underexplored.

Importantly, most studies to date have focused on intestinal or fecal bacterial communities, whereas the nasal microbiota has been largely overlooked. Given its role in respiratory health and disease susceptibility, investigating whether stocking density also influences the nasal microbial communities is warranted. Since stocking density housing conditions are also linked to increased respiratory disease risk in swine production [47, 48], characterizing changes in the nasal microbiota may provide critical insights into the broader systemic effects of crowded conditions.

Understanding the relationships among stocking density, microbial communities, and host physiology is essential for optimizing pig productivity. In this study, we investigated the effects of high stocking density on growth performance, stress biomarkers, nasal and fecal bacterial communities, microbial metabolite profiles, and predicted microbial functions in finishing pigs. We further explored potential associations between microbial changes, immune responses, and growth outcomes. With the aim of providing new perspectives into how pig management practices influence microbiota and overall pig health in commercial production systems, we hypothesize that high stocking density exerts systemic effects on the host by reshaping microbial communities and their metabolic outputs, which can potentially compromise immune function and growth outcomes.

## Methods

This study was conducted using institutional animal care and use of practices in accordance and as approved by the Animal Care Committee of Dankook University Cheonan Campus in South Korea (approval number: DK-2-2426).

### Animals, housing, and experimental design

A total of 98 pigs [(Landrace × Yorkshire) × Duroc], aged 21 weeks and of random sex, with an average initial body weight of 87.39 ± 0.18 kg, were housed in a piggery with a pen area of 2.91 m^2^ (Supplementary Fig. S1). All pens were in the same environmentally controlled barn with slatted floors and standard ventilation system. Prior to the start of the study, pigs were managed under the same feeding, housing, and health care routines to minimize environmental and management-related variability. Animals were randomly selected from multiple litter to avoid litter-specific bias. After 21 weeks, pigs were balanced across treatment groups to ensure comparable initial body weight, which were then assigned to two stocking density groups in a completely randomized block design: a control (CON) group with a space allowance of 0.97 m^2^ per pig (*k* = 0.049) comprising of 3 pigs per pen in 16 replicates (*n* = 48), and a high stocking density (HD) group with a space allowance of 0.58 m^2^ per pig (*k* = 0.029) in 10 replicates of 5 pigs per pen (*n* = 50). The HD group represents 60% of the CON group’s space allowance, which was designed based on the minimum space allowance required for pigs weighing 60–110 kg (0.8 m^2^/pig) as per the Enforcement Decree of the Livestock Industry Act in South Korea [28] and the minimum space allowance coefficient for pigs weighing up to 110 kg (*k* = 0.047) prescribed by the European Food Safety Authority [7]. For 4 weeks, the pigs were given *ad libitum* access to water via a nipple waterer and fed through a one-sided self-feeder. A basal feed diet (Supplementary Table S1) was formulated to meet the nutritional requirements of finishing pigs [33]. Blood sampling, fecal collection, and coughing index observations were conducted starting at 7:00 AM, before feed was offered.

### Growth performance

Each animal’s body weight was recorded individually at the beginning and end of the 4-week experimental period. The average daily gain (ADG), average daily feed intake (ADFI), and feed conversion ratio (FCR) were calculated. Feed intake was determined by subtracting the remaining feed from the feed offered at the time of initial weighing, and FCR was calculated by dividing ADFI by ADG.

### Nutrient digestibility

After the 4-week experimental period, chromium oxide (Cr₂O₃) was incorporated into the diet at a 0.5% inclusion rate as an indigestible marker for 7 days. A total of 20 representative pigs per group were randomly sampled from the pens. Fecal samples were collected using the rectal massage technique. The collected feces were dried in an oven at 60 °C for 72 h, ground using a Wiley mill, and subsequently analyzed for nutrient composition. The proximate composition of the feed and the chromium content (used as a marker) were determined [1].

### Blood parameter

Similarly, after the 4-week experimental period, 5 mL blood was drawn from the jugular vein of each animal into a vacuum tube (Becton Dickinson Vacutainer Systems, Franklin Lakes, NJ, USA). As with nutrient digestibility, the same 20 representative pigs per group were selected from the pens. Blood samples were centrifuged at 845 × *g* for 15 min to separate serum. Serum cortisol levels were measured using a Cortisol Enzyme-Linked Immunosorbent Assay (ELISA) kit (MyBioSource, CA, USA). Additionally, tumor necrosis factor-alpha (TNF-α) and interleukin-6 (IL-6) concentrations were quantified using TNF-α and IL-6 ELISA kits, respectively (MyBioSource, CA, USA).

### Coughing index

Coughing was monitored daily for 30 min. The coughing index was determined by scoring the coughs and calculating a weekly average: score 0 = no cough; score 1 = single cough with low sound intensity; and score 2 = multiple loud, persistent coughs. All coughing observations were carried out by a single trained observer to ensure consistency in scoring.

### Microbiota sample collection

In addition to blood samples, nasal and fecal samples were collected from all pigs for analysis after the 4-week experimental period. Fresh fecal samples were obtained directly from the rectum. Nasal samples were collected using rayon-tipped swabs inserted at an angle of approximately 45 °, 5–8 cm deep into the nostril. The swab was rotated three times each clockwise and counterclockwise to ensure complete contact with the nasal cavity. The same swab was used to sample the other nostril following the same procedure before being placed back into the collection tube. Care was taken to avoid contact with environmental debris during swabbing. All samples were transported under refrigerated conditions and stored at − 80 °C until analysis. For nasal swabs, DNA extraction was aseptically performed immediately upon arrival at the laboratory and was completed on the same day of collection to minimize contamination and DNA degradation.

### DNA extraction and 16S rRNA sequencing

To prepare nasal swab samples for DNA extraction, the swab tip was carefully cut into a 15-mL conical tube containing 1 mL of 1× phosphate-buffered saline (PBS) solution. An additional 1 mL of 1× PBS was used to wash the inside of the collection tube, which was then collected in the same conical tube. Tubes were vortexed vigorously for 1 min to release the contents from the swabs. The swab was then removed aseptically, and the tube was centrifuged at 15,000 × *g* at 4 °C for 15 min. The resulting pellets were collected and subjected to DNA extraction. Fecal samples (0.25 g) were aseptically collected in bead-beating tubes and used for DNA extraction. Genomic DNA from fecal samples was extracted using the QIAamp PowerFecal^®^ Pro DNA Kit (Qiagen, Hilden, Germany), following the manufacturer’s instructions. Based on prior studies applying fecal DNA extraction kits to nasal and respiratory samples in humans and swine [9, 24, 44, 60, 65], as well as our in-house laboratory validation, the same kit was used to extract genomic DNA from nasal swabs. This approach minimized contamination risk while ensuring reliable recovery of microbial DNA from low-biomass samples. The concentration and purity of genomic DNA were assessed using a SpectraMax M2 spectrophotometer (Molecular Devices, CA, USA). Sequencing libraries were prepared and the full-length 16 S rRNA gene (V1-V9) was sequenced using PacBio Sequel (Pacific Bioscience, CA, USA) by CJ BioScience, Inc. (Seoul, Republic of Korea).

### Analyses of microbial diversity, structure, composition and predicted functions

Raw 16S rRNA sequence data were processed using Quantitative Insights into Microbial Ecology (QIIME 2) Amplicon Distribution pipeline [4]. Denoising, removal of primers and adapters, sequence quality control, and feature table construction were performed with DADA2 [5] via the ‘q2-dada2’ plugin. In addition to the default DADA2 filtering parameters, we applied a customized quality-control pipeline optimized for low-biomass samples. This included amplicon length filtering to retain only sequences matching the expected length of the targeted 16S region, removal of unassigned ASVs, and filtering of extremely low-abundance features prior to downstream taxonomic and diversity analyses. The phylogenetic tree was constructed from the feature table using ‘q2-phylogeny.’ Sequencing depth was evaluated through alpha-rarefaction using ‘q2-diversity,’ followed by an analysis of alpha- and beta-diversity metrics. Alpha-diversity metrics were described using Shannon entropy and Simpson indices, whereas the Bray–Curtis dissimilarity distance matrix was used for beta-diversity analysis through principal coordinate analysis (PCoA) to capture abundance-driven compositional dissimilarities among groups. The feature classifier was built based on SILVA SSU-NR99 16S database version 138.1 [41] and trained with a ‘q2-feature-classifier’ to determine the relative abundance of the microbial community at different taxonomic ranks. Differential abundance analysis for identifying taxonomic markers with relative abundance ≥ 0.01% was then performed using Linear Discriminant Analysis Effect Size (LEfSe) [43], ensuring that only robust and biologically interpretable features were evaluated. Plots for visualizing alpha- and beta-diversity metrics, taxonomic relative abundance, and taxonomic markers were generated in the R program using the ‘ggplot2’ package [55, 42]. Metabolic functional pathways were predicted using PICRUSt2 (Phylogenetic Investigation of Communities by Reconstruction of Unobserved States) [12] and annotated using the Kyoto Encyclopedia of Genes and Genomes (KEGG). Functional markers for predicted metabolism-related KEGG pathways were analyzed and visualized using STAMP (Statistical Analysis of Metagenomic Profiles) version 2.1.3 [36]. Correlations were analyzed using log_(x+1)_-normalized data, clustered based on the Pearson correlation coefficient (*r*), and visualized with the ‘pheatmap’ package in R [21, 42].

### Determination of gut microbiota-derived metabolite concentrations using high-performance liquid chromatography

Fecal samples were prepared for the determination of gut microbiota-derived metabolite concentrations using high-performance liquid chromatography (HPLC) [16, 35, 51]. Briefly, feces (0.5 g) were suspended in 1 mL of 5 mM sulfuric acid (H_2_SO_4_) in a 2-mL tube and placed on ice. Samples were vortexed thrice for 1 min, with 1-min intervals on ice, and then centrifuged at 15,000 × *g* at 4 °C for 10 min. The supernatant was collected, filtered through 0.20 μm PTFE-W syringe filter (BioFACT, Republic of Korea) into 2-mL clear glass screw vials with silicon septum (Thermo Fisher Scientific, MA, USA), and kept at 4 °C. The concentration (in mM/g of dry feces weight) of lactate and short-chain fatty acids (SCFA), particularly acetate, propionate, and butyrate, were quantified using an Agilent Infinity 1260 HPLC System (Agilent, CA, USA) equipped with refractive index and ultra-violet (UV; λ = 210 nm) detectors. A Carbomix H-NP 10:8% column (300 × 7.8 mm, 10 μm non-porous; Sepax Technologies, Inc., DE, USA) was used with 5 mM H_2_SO_4_ as the mobile phase. Samples (10 µL) were injected via autosampler, maintained at 10 °C, and analyzed at a column temperature of 65 °C with a flow rate of 0.8 mL/min for 55 min.

### Statistical analyses

Statistical analyses were conducted with pens treated as experimental units. Data normality was assessed using the Shapiro–Wilk test in GraphPad Prism version 9.0.0 (GraphPad, MA, USA). Growth performance, blood parameters, and coughing index were confirmed to meet the assumptions of normality and homogeneity of variance, and were subsequently analyzed using independent *t*-tests in SAS (SAS Institute Inc., Cary, NC, USA). Group comparisons for alpha-diversity metrics, relative taxonomic abundances, and fecal metabolite concentrations were performed using the Mann–Whitney U test. Beta-diversity was assessed using principal coordinate analysis (PCoA) based on Bray–Curtis dissimilarity. Differences between groups were evaluated using permutational multivariate analysis of variance (PERMANOVA) with 999 permutations, and the effect of stocking density on bacterial communities was quantified using the R^2^. Statistical significance was set at *P* < 0.05. Differentially abundant taxa were identified using LEfSe, with Wilcoxon and Kruskal–Wallis test cutoffs of *P* < 0.05, a linear discriminant analysis (LDA) score of ≥ 2, and a stringency level of 2 to minimize false positives. Welch’s *t*-test with Storey FDR correction (adjusted *P*-value: *q* < 0.05) was used to analyze the relative abundances of predicted metabolism-related KEGG functional markers. Pearson’s correlation analyses were conducted to assess associations between microbial and physiological data, with thresholds for significance set at *P* < 0.05, *P* < 0.01, and *P* < 0.001. Fecal metabolite data were analyzed in GraphPad and visualized in R, whereas all other microbiota analyses and visualizations were conducted in R.

## Results

### Growth performance and nutrient digestibility of pigs

Table 1 summarizes the effects of stocking density on pig productivity. At the end of the 4-week experimental period, a significant decrease in the final body weight (*P* = 0.048) was observed in pigs housed under high stocking density condition. Moreover, over the entire experimental period, a significant reduction in the ADG (*P* = 0.035) and ADFI (*P* = 0.048) of finishing pigs in high stocking density was found. These results suggest that lower stocking density may be associated with improved growth performance, whereas increased crowding caused by high stocking density may lead to impaired growth and productivity.

**Table 1:** Effect of stocking density on growth performance in finishing pigs

Items	CON	HD	SEM^1^	*P*-value^2^
Body weight, kg				
Initial	87.39	87.39	0.18	0.998
Final	108.60^a^	107.31^b^	0.42	0.048
Overall				
ADG, g	758^a^	711^b^	13	0.035
ADFI, g	2,266^a^	2,155^b^	35	0.048
FCR	2.993	3.031	0.019	0.156

Abbreviations: CON = control (*n* = 48); HD = high stocking density (*n* = 50); ADG = average daily gain; ADFI = average daily feed intake; FCR = feed conversion ratio

^1^ Standard error of means

^2^ Statistical significance determined using *t*-test

^a, b^ Means in the same row with different superscripts differ significantly (*P* < 0.05)

The effects of stocking density on nutrient digestibility are presented in Table 2. No significant differences were detected between the groups in dry matter, nitrogen, or energy digestibility (*P* > 0.05 for all parameters). Different stocking densities did not affect the apparent total tract digestibility of nutrients.

**Table 2:** Effect of stocking density on nutrient digestibility in finishing pigs

Items, %	CON	HD	SEM^1^	*P*-value^2^
Dry matter	73.31	72.76	0.31	0.186
Nitrogen	69.43	68.85	0.22	0.118
Energy	72.04	71.55	0.33	0.308

Abbreviations: CON = control (*n* = 20); HD = high stocking density (*n* = 20)

^1^ Standard error of means

^2^ Statistical significance determined using *t*-test

### Blood parameters and coughing index of pigs

Table 3 shows the effects of the different stocking densities on the blood parameters measured after the 4-week experimental period. Under high stocking density, cortisol levels were significantly increased (*P* = 0.037).

**Table 3:** Effect of stocking density on blood profile in finishing pigs

Items	CON	HD	SEM^1^	*P*-value^2^
Cortisol, µg/dL	1.23^b^	1.48^a^	0.07	0.037
TNF-α, pg/mL	115.34	116.73	1.01	0.462
IL-6, pg/mL	83.39	84.97	0.97	0.391

Abbreviations: CON = control (*n* = 20); HD = high stocking density (*n* = 20); TNF-α = tumor necrosis factor-α; IL-6 = interleukin-6

^1^ Standard error of means

^2^ Statistical significance determined using *t*-test

^a, b^ Means in the same row with different superscripts differ significantly (*P* < 0.05)

The data in Table 4 demonstrate that the coughing index differed markedly between the groups. Throughout the experimental period, pigs in the HD group exhibited a significantly higher coughing index than those in the CON group (*P* < 0.001).

**Table 4:** Effect of stocking density on coughing score of finishing pigs^1^

Week	CON	HD	SEM^2^	*P*-value^3^
Week 1	0.01^b^	0.09^a^	0.01	< 0.001
Week 2	0.07^b^	0.23^a^	0.02	< 0.001
Week 3	0.17^b^	0.51^a^	0.04	< 0.001
Week 4	0.39^b^	0.89^a^	0.07	< 0.001

Abbreviations: CON = control (*n* = 48); HD = high stocking density (*n* = 50)

^1^ Coughing index: 0 = not coughing; 1 = one-off and quiet cough; 2 = continuous and loud cough

^2^ Standard error of means

^3^ Statistical significance determined using *t*-test

^a, b^ Means in the same row with different superscripts differ significantly (*P* < 0.05)

### Pig nasal and fecal microbial diversity and structure

Sequencing of the full-length 16S rRNA gene yielded 2,082,201 and 978,212 16S total raw reads for the nasal and fecal samples of finishing pigs, respectively. After filtering out chimeric and low-quality sequences, 813,875 and 106,706 total valid reads were retained for the nasal (average length = 1,444 bp) and fecal samples (average length = 1,459 bp), respectively. All 98 samples passed quality control and were included in the downstream analyses. Following denoising with DADA2, the final sequencing depth per sample was 2,208 reads for nasal samples and 254 reads for fecal samples (Supplementary Table S2). Both the CON and HD groups in nasal and fecal samples achieved a Good’s coverage of 100%, confirming the sufficiency of amplicon sequencing depths for downstream analyses. Additionally, alpha-rarefaction curves based on observed features further supported that the sequencing depth were adequate to capture the microbial diversity in all samples (Supplementary Fig. S2).

**Fig. 1:**
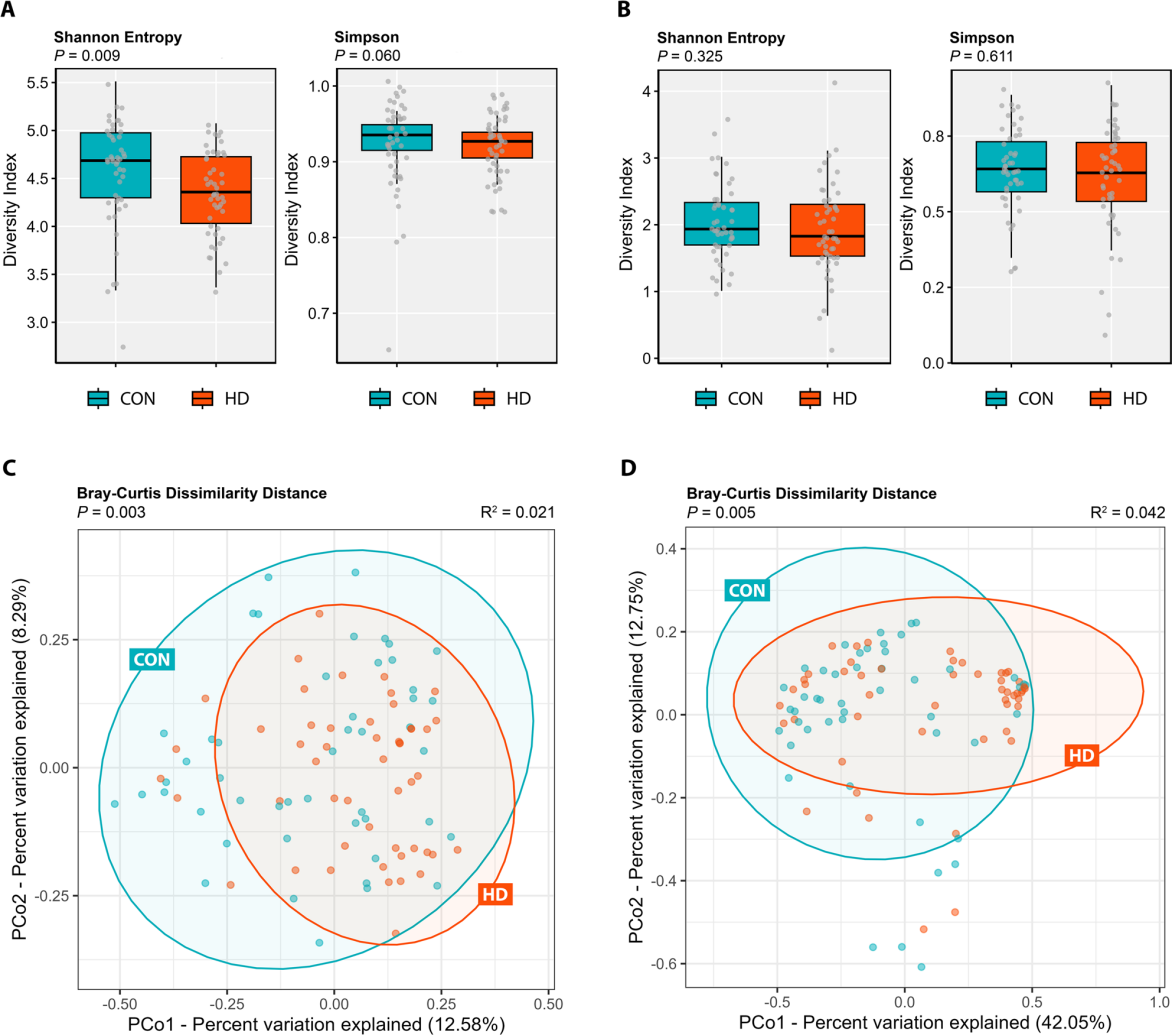
Changes in the bacterial community structure of finishing pigs under high stocking density. Comparison of Shannon entropy and Simpson indices of nasal (**A**) and fecal (**B**) samples. Principal coordinate analysis (PCoA) plots of beta-diversity index based on Bray-Curtis dissimilarity distance matrix of nasal (**C**) and fecal (**D**) samples. Significance was calculated using Mann-Whitney U test and PERMANOVA (*P* < 0.05) for alpha- and beta-diversity indices, respectively

To investigate the influence of high stocking density on the diversity and structure of the nasal and fecal microbiota of finishing pigs, the alpha- and beta-diversity indices were measured (Fig. 1; Supplementary Table S3). The Shannon entropy (*P* = 0.009) alpha-diversity indices in the HD group were significantly lower than those in the CON group for nasal samples (Fig. 1A). A reduction in alpha-diversity indices was also observed in fecal samples, but these were not significant (*P* > 0.05) (Fig. 1B). The Simpson index showed no significant difference between the HD and CON groups for both nasal and fecal microbiota (*P* > 0.05). Additionally, beta-diversity analyses using PCoA plots based on the Bray–Curtis dissimilarity matrix revealed statistically significant differences in bacterial community structure between groups for both nasal (*P* = 0.003) and fecal (*P* = 0.005) samples (Fig. 1C and D, respectively). Although the R² values indicate that stocking density explains only a small proportion of the total variation (nasal = 2.1%; fecal = 4.2%), samples from the HD group still clustered distinctly from those of the CON group. These findings indicate that high stocking density induces measurable but modest compositional shifts in the nasal and fecal bacterial communities, even in cases where within-sample diversity shows limited or no statistical differences.

### Pig nasal and fecal microbiota composition

**Fig. 2:**
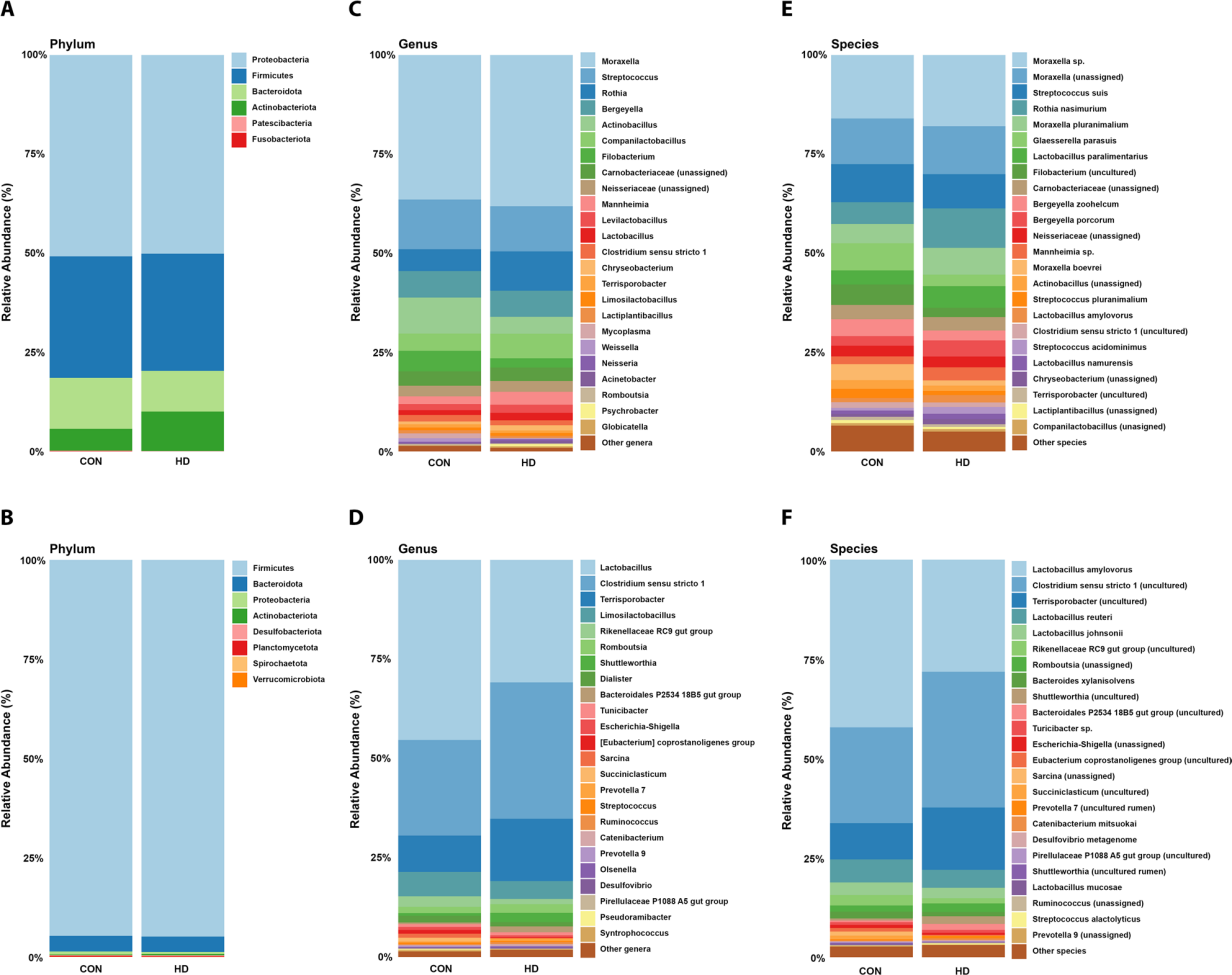
Changes in bacterial composition of finishing pigs associated with high stocking density. Relative abundance (%) at phylum (**A** and **B**), genus (**C** and **D**), and species (**E** and **F**) levels in the nasal and fecal samples, respectively, emphasizing the top 24 taxa at the genus and species levels

The current study also examined the effect of high stocking density on the bacterial composition in nasal and fecal samples of finishing pigs (Supplementary Table S4 and S5). At the phylum level, Proteobacteria was the dominant taxonomic group in the nasal bacterial community of finishing pigs, accounting for 50.77 ± 2.058% in CON and 50.12 ± 1.602% in HD (Fig. 2A). This was followed by Firmicutes (30.66 ± 1.940% in CON and 29.56 ± 2.135% in HD) and Bacteroidota (previously Bacteroidetes) (12.89 ± 1.379% in CON and 10.26 ± 1.266% in HD), whereas Patescibacteria and Fusobacteria accounted for less than 0.1% in both groups. Notably, Actinobacteriota (previously Actinobacteria) showed a significant increase in relative abundance in the HD group (9.96 ± 1.112%) compared with the CON group (5.50 ± 0.758%; *P* = 0.005). Firmicutes dominated the fecal microbiota, accounting for 94.66 ± 1.420% of the CON group and 94.87 ± 1.328% of the HD group (Fig. 2B). The phylum Bacteroidota accounted for 3.94 ± 1.229% in the CON group and 3.86 ± 1.085% in the HD group. The remaining 1.40% of the CON group and 1.27% of the HD group comprised Proteobacteria, Actinobacteriota, Desulfobacteria, Planctomycetota, Spirochaetota, and Verrucomicrobiota, with Verrucomicrobiota present only in the HD group at 0.02 ± 0.018%.

At the genus level (Fig. 2C), *Moraxella* was the most abundant taxon, comprising 36.44 ± 2.333% and 38.14 ± 1.947% of the nasal microbial community in CON and HD groups, respectively. This was followed by *Streptococcus* (12.57 ± 0.840% in CON and 11.40 ± 1.208% in HD), *Rothia* (5.50 ± 0.758% in CON and 9.94 ± 1.109% in HD), *Bergeyella* (6.68 ± 1.127% in CON and 6.58 ± 1.099% in HD), *Actinobacillus* (9.09 ± 1.413% in CON and 4.26 ± 0.980% in HD) and *Companilactobacillus* (4.37 ± 1.243% in CON and 6.20 ± 1.391% in HD). Several taxa were found to be significantly more abundant in HD than in CON, including *Rothia* (*P* = 0.005) and *Psychrobacter* (*P* = 0.026), with *Psychrobacter* being present only in the HD group (0.55 ± 0.312%). Conversely, nasal populations of *Actinobacillus* (*P* = 0.001), *Filobacterium* (5.16 ± 0.949% in CON vs. 2.33 ± 0.693% in HD; *P* = 0.001), and *Mycoplasma* (1.21 ± 0.325% in CON vs. 0.17 ± 0.071% in HD; *P* < 0.001) were significantly reduced in pigs at high stocking density. In the fecal microbial community (Fig. 2D), *Lactobacillus* was the most abundant genus in the CON group (45.35 ± 3.801%), followed by *Clostridium sensu stricto* 1 (24.08 ± 2.773%), and *Terrisporobacter* (9.13 ± 1.246%). In contrast, the abundance of *Lactobacillus* was significantly decreased in the HD group (30.84 ± 4.336%; *P* = 0.019), whereas *Clostridium sensu stricto* 1 and *Terrisporobacter* were significantly enriched in the HD group (34.33 ± 3.277%; *P* = 0.026 and 15.69 ± 1.808%; *P* = 0.010, respectively). Moreover, significant reductions in the populations of *Succiniclasticum* (0.92 ± 0.442% in CON to 0.04 ± 0.041% in HD; *P* = 0.042) and *Sarcina* (1.04 ± 0.578% in CON; *P* = 0.020) were observed in the HD group.

The most abundant species in the nasal microbiota was *Moraxella* sp., accounting for 16.02 ± 1.757% in CON and 17.96 ± 1.620% in HD, followed by an unassigned *Moraxella* (11.51 ± 1.646% in CON and 12.09 ± 1.083% in HD), *Streptococcus suis* (9.60 ± 0.871% in CON and 8.64 ± 0.985% in HD), *Rothia nasimurium* (5.48 ± 0.759% in CON and 9.94 ± 1.109% in HD), *Moraxella pluranimalium* (4.89 ± 0.911% in CON and 6.77 ± 1.138% in HD), and *Glaesserella parasuis* (6.85 ± 1.083% in CON and 2.91 ± 0.564% in HD) (Fig. 2E). The abundance of several species was significantly enriched in HD group, including *R. nasimurium* (*P* = 0.005) and *Psychrobacter faecalis* (0.51 ± 0.285% in HD; *P* = 0.026). However, a reduction in the abundance of *G. parasuis* (*P* = 0.002), uncultured *Filobacterium* (*P* = 0.001), *Moraxella boevrei* (4.02 ± 1.058% in CON vs. 1.32 ± 0.362% in HD; *P* = 0.007), unassigned *Actinobacillus* (2.22 ± 0.599% in CON vs. 1.35 ± 0.481% in HD; *P* = 0.010), *Streptococcus pluranimalium* (2.28 ± 0.520% in CON vs. 1.06 ± 0.279% in HD; *P* = 0.024), *Mycoplasma hyorhinis* (0.95 ± 0.315% in CON vs. 0.15 ± 0.071% in HD; *P* = 0.001), and *Mesomycoplasma flocculare* (0.26 ± 0.118% in CON vs. 0.02 ± 0.013% in HD; *P* = 0.017) was observed in the nasal microbiota of HD group. Additionally, the nasal populations of *Lactobacillus songhuajiangensis* (0.11 ± 0.048%; *P* = 0.001), *Lactobacillus heilongjiangensis* (0.02 ± 0.012%; *P* = 0.039), and *Bacteroides xylanisolvens* (0.05 ± 0.019%; *P* = 0.001) were completely depleted in the HD group. In the fecal microbiota, *L. amylovorus* was the most abundant species across all groups (42.19 ± 4.023% in CON; 28.21 ± 4.333% in HD) (Fig. 2F). However, the fecal abundance of *L. amylovorus* significantly decreased in the HD group (*P* = 0.021). Together with *L. amylovorus*, significant reductions in the populations of *Lactobacillus johnsonii* (3.16 ± 1.537% to 2.64 ± 1.725%; *P* = 0.035) and uncultured *Succiniclasticum* (0.92 ± 0.442% to 0.04 ± 0.041%; *P* = 0.042) were also observed in the HD group, while an unassigned *Sarcina* was not detected in the fecal microbial community of pigs in the HD group but present in the CON group (1.04 ± 0.578%; *P* = 0.20). In contrast, uncultured species of *Clostridium sensu stricto* 1 and *Terrisporobacter* were significantly more abundant in the HD group (34.12 ± 3.258%; *P* = 0.028 and 15.69 ± 1.808%; *P* = 0.010, respectively) than in the CON group (24.07 ± 2.773% and 9.13 ± 1.246%, respectively).

**Fig. 3:**
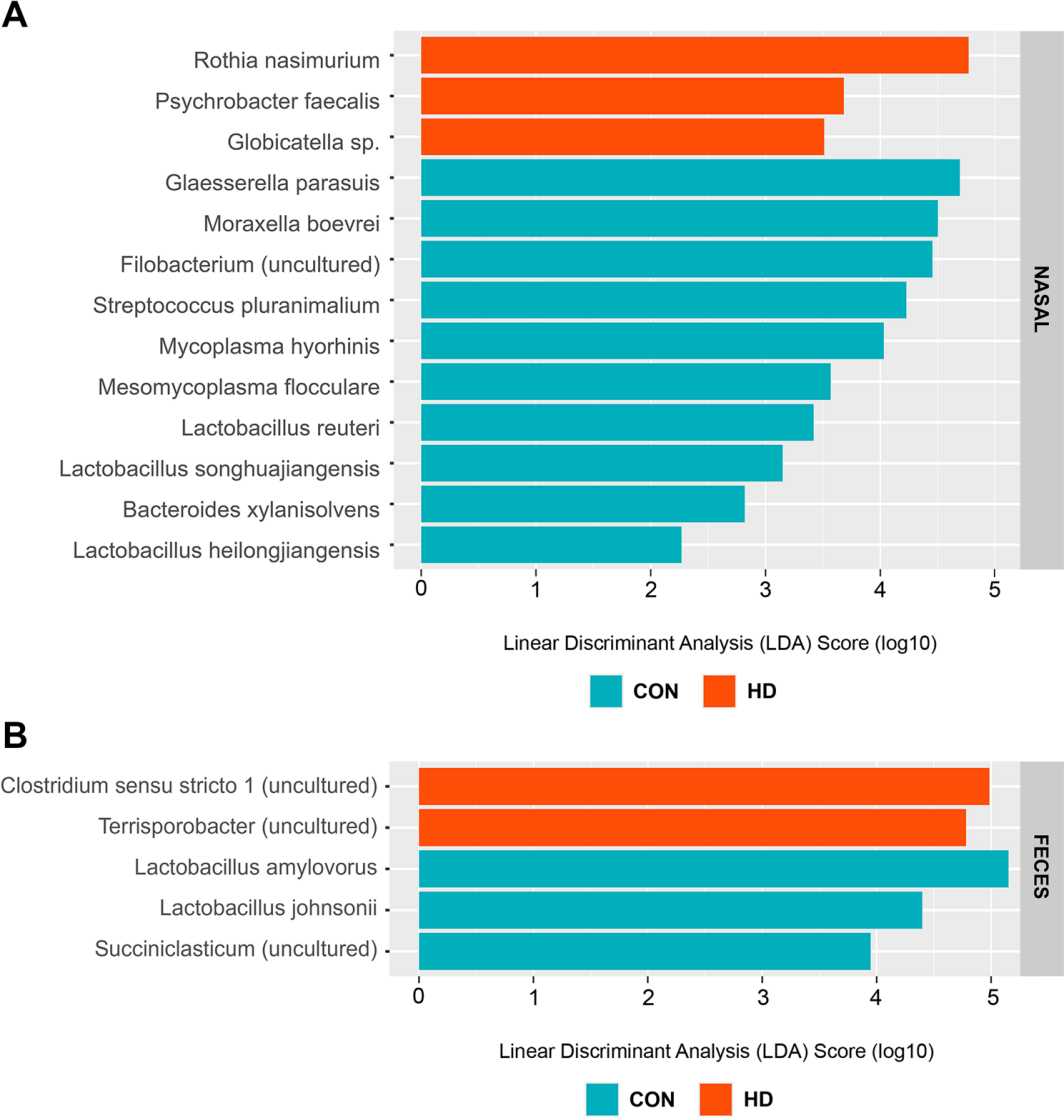
Species-level taxonomic markers in the nasal (**A**) and fecal (**B**) microbiota of finishing pigs. Statistical significance set at *P* < 0.05 determined using differential abundance analysis (LEfSe)

To identify the significant species-level taxonomic markers in the nasal and fecal bacterial communities of each group (*P* < 0.05), differential abundance analysis was conducted using LEfSe. In the HD group, significant enrichment was observed for *R. nasimurium*, *P. faecalis*, and *Globicatella* sp. in the nasal microbiota (Fig. 3A) and for uncultured *Clostridium sensu stricto* 1 and *Terrisporobacter* in the fecal microbiota (Fig. 3B). Conversely, the CON group was characterized by a significant enrichment of *G. parasuis*, *M. boevrei*, uncultured *Filobacterium*,* S. pluranimalium*, *M. hyorhinis*, *M. flocculare*, *L. reuteri*, *L. songhuajiangensis*, *B. xylanisolvens*, and *L. heilongjiangensis* in the nasal microbiota (Fig. 3A), and *L. amylovorus*,* L. johnsonii*, and uncultured *Succiniclasticum* in the fecal microbiota (Fig. 3B).

### Predicted microbial functions of pigs

**Fig. 4:**
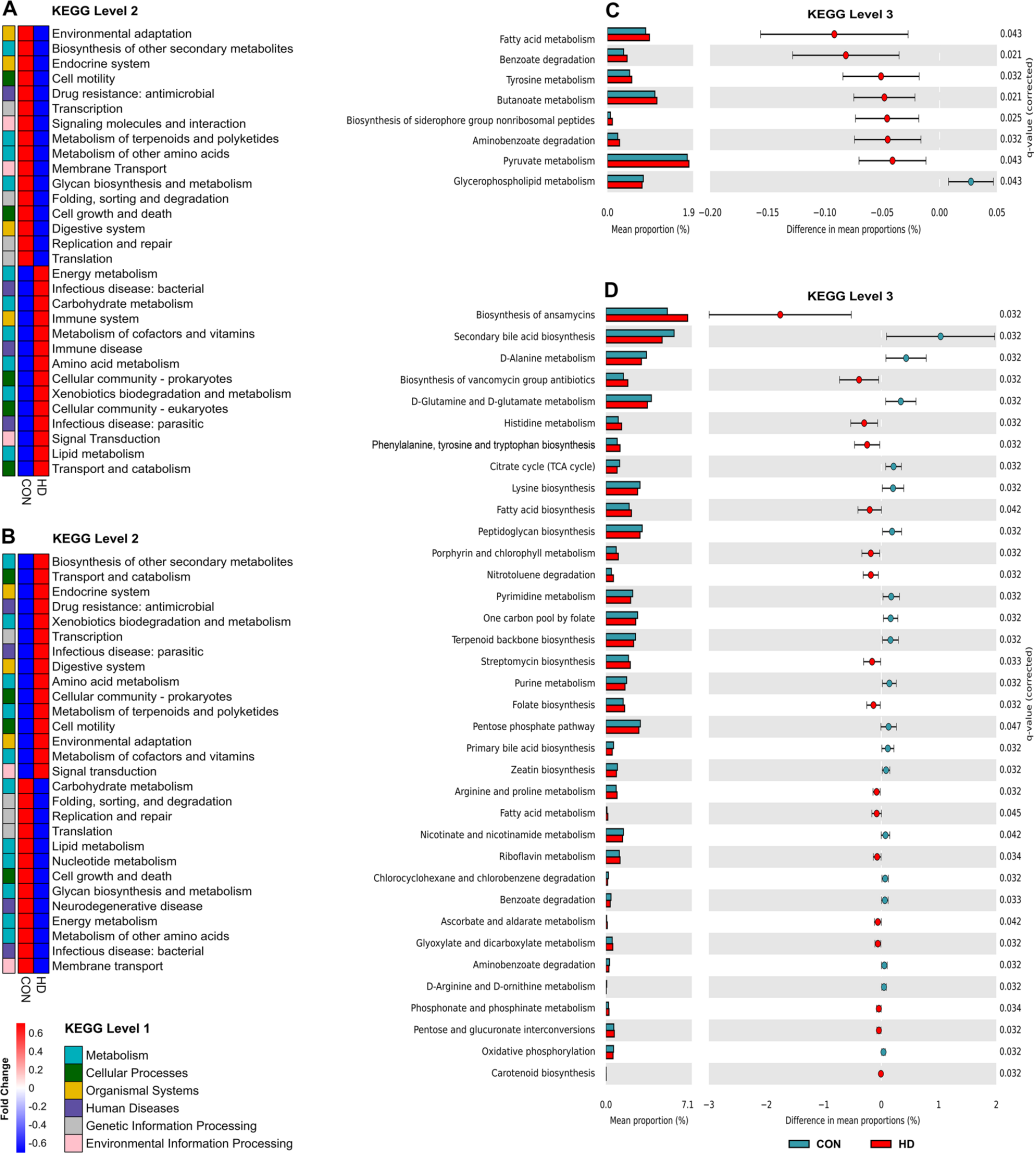
Shifts in predicted nasal and fecal microbial functions of finishing pigs under high stocking density. Fold-change in the relative abundance of KEGG functional pathways (Level 1 and 2) between CON and HD groups of the nasal (**A**) and fecal (**B**) samples. Significant differences in the mean proportion (%) of the relative abundances of predicted metabolism-related KEGG pathways (Level 3) in the nasal (**C**) and fecal (**D**) samples determined using Welch’s *t*-test with Storey FDR correction (*q* < 0.05)

In the nasal microbiota of pigs under HD group (Fig. 4A), pathways related to immune and infectious diseases, including bacterial and parasitic infections, as well as immune system functions and signal transduction, were enriched. Conversely, decreased pathway abundance for biosynthesis of other secondary metabolites, metabolism of terpenoids and polyketides, metabolism of other amino acids, glycan biosynthesis and metabolism, and antimicrobial drug resistance were observed.

High stocking density also reshaped the predicted functions of the fecal microbiota (Fig. 4B), leading to the enrichment of pathways related to the biosynthesis of secondary metabolites, xenobiotics biodegradation, amino acid metabolism, and the metabolism of terpenoids, polyketides, and cofactors. Conversely, pathways involved in energy, carbohydrate, lipid, nucleotide, glycan biosynthesis, and other amino acid metabolism were diminished. In terms of immune function, high stocking density resulted in an increase in pathways associated with antimicrobial resistance and parasitic diseases, while pathways linked to bacterial infectious diseases and neurodegenerative diseases were reduced.

Significant differences in the predicted metabolism-related KEGG functional pathways were observed in the microbial communities of pigs under high stocking density. In the nasal microbiota (Fig. 4C), high stocking density resulted in significant increases (*q* < 0.05) in the mean proportions (%) of pathways involved in carbohydrate metabolism (butanoate metabolism and pyruvate metabolism), amino acid metabolism (tyrosine metabolism), and lipid metabolism (fatty acid metabolism). Additionally, other pathways, including benzoate metabolism, aminobenzoate degradation, and the biosynthesis of siderophore group nonribosomal peptides, were significantly enriched under high stocking density (*q* < 0.05). In contrast, glycerophospholipid metabolism was significantly reduced under high stocking density (*q* < 0.05).

In the fecal microbiota, high stocking density was associated with significant increases (*q* < 0.05) in the mean proportions (%) of pathways related to carbohydrate (pentose and glucoronate interconversions, ascorbate and aldarate metabolism, and glyoxylate and dicarboxylate metabolism), amino acid metabolism (arginine and proline metabolism, histidine metabolism, and phenylalanine, tyrosine, and tryptophan metabolism), co-factor and vitamin metabolism (folate biosynthesis, riboflavin metabolism, and porphyrin and chlorophyll metabolism), and lipid metabolism (fatty acid biosynthesis and fatty acid metabolism) (Fig. 4D). Additionally, other metabolic pathways were significantly enriched under high stocking density (*q* < 0.05), including carotenoid biosynthesis, nitrotoluene degradation, streptomycin biosynthesis, phosphonate and phosphinate metabolism, biosynthesis of vancomycin group antibiotics, and biosynthesis of ansamycins. Conversely, several metabolic pathways were significantly reduced (*q* < 0.05) under high stocking density. These reductions included pathways related to carbohydrate metabolism (citrate cycle and pentose phosphate pathway), amino acid metabolism (D-arginine and D-ornithine metabolism and lysine metabolism), energy metabolism (oxidative phosphorylation), co-factor and vitamin metabolism (one carbon pool by folate and nicotinate and nicotinamide metabolism), and lipid metabolism (primary bile acid biosynthesis and secondary bile acid biosynthesis). Other pathways significantly reduced under high stocking density (*q* < 0.05) included benzoate degradation, chlorocyclohexane and chlorobenzene degradation, aminobenzoate degradation, purine metabolism, terpenoid backbone biosynthesis, pyrimidine metabolism, D-alanine metabolism, D-glutamine and D-glutamate metabolism, zeatin biosynthesis and peptidoglycan biosynthesis.

### Pig gut microbiota-derived metabolite concentrations

**Fig. 5:**
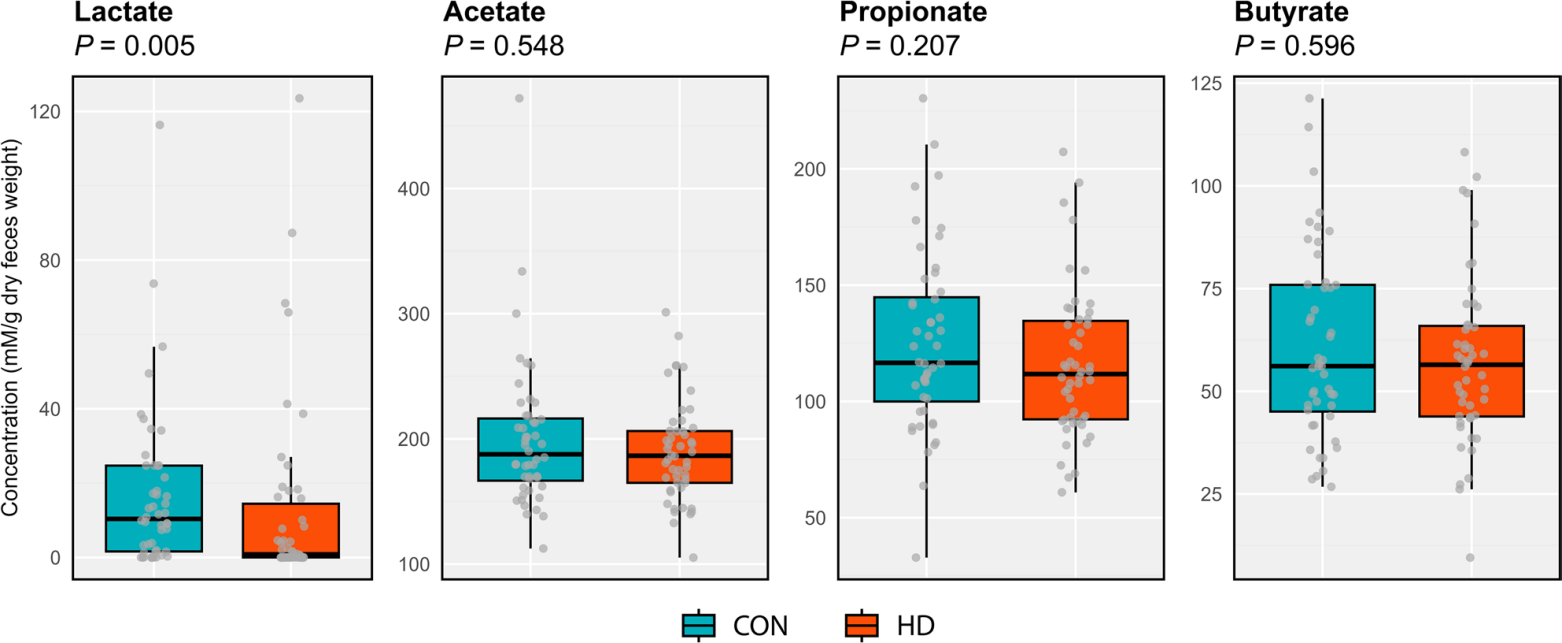
Changes in gut microbiota-derived metabolite concentration of finishing pigs associated with high stocking density. Comparison of fecal lactate and SCFA (acetate, propionate, and butyrate) concentration (mM/g dry feces weight) in CON and HD groups using Mann-Whitney U test (*P* < 0.05)

To investigate the effect of high stocking density on gut microbiota-derived metabolites, the concentrations of lactate and SCFA (acetate, propionate, and butyrate) in the fecal samples of finishing pigs were measured (Fig. 5; Supplementary Table S6). The fecal lactate concentration was significantly lower in the HD group than in the CON group (*P* = 0.005). Meanwhile, the fecal levels of acetate, propionate, and butyrate were also lower in the HD group than in the CON group, but not significantly.

### Associations of pig microbiota with growth performance and blood parameters

**Fig. 6:**
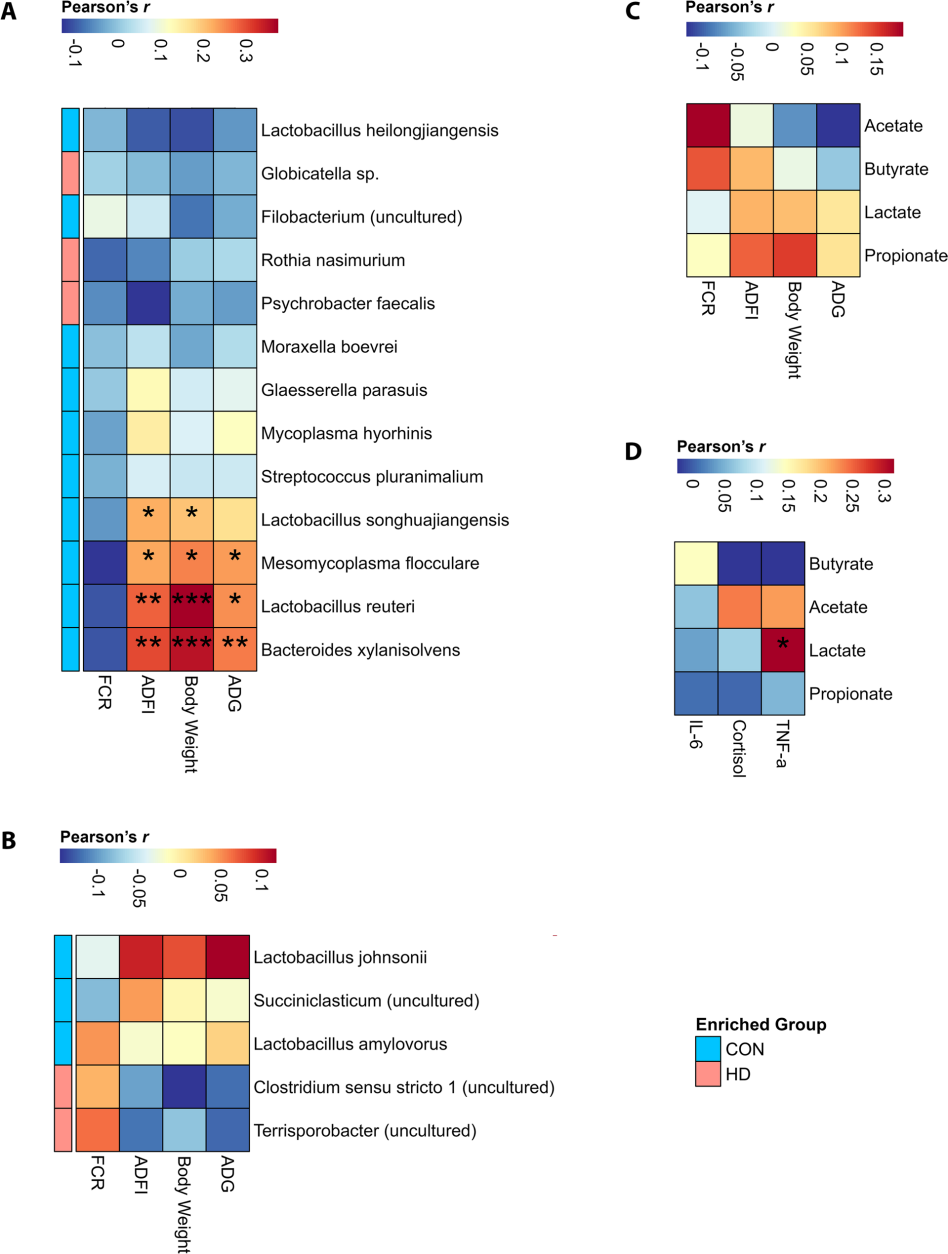
Pearson correlation heatmap of physiological and microbial shifts in finishing pigs under high stocking density. Panel shows associations between growth performance and the taxonomic markers of the nasal (**A**) and fecal (**B**) microbiota, as well as between gut microbiota-derived metabolites and growth performance (**C**) and blood parameters (**D**). Data was normalized through log(_x+1_) transformation and clustered based on Pearson’s *r* values. The *P*-values were denoted as *, **, and *** for *P* < 0.05, *P* < 0.01, and *P* < 0.001, respectively. Abbreviations: FCR = feed conversion ratio; ADFI = average daily feed intake; body weight = final body weight (in kg); ADG = average daily gain; TNF-α = tumor necrosis factor-α; IL-6 = interleukin-6

The relationship between the nasal microbiota and growth performance was first assessed. Correlation heatmaps showed negative associations between nasal microbial taxa enriched under high stocking density and all growth performance metrics, particularly ADG, ADFI, FCR, and final body weight, although these were not statistically significant (Fig. 6A). In contrast, several taxa in the nasal microbial community enriched in the CON group were significantly positively correlated with growth performance. Notably, ADFI and final body weight were positively correlated with *L. reuteri* (*P* = 0.005 and *P* < 0.001), *B. xylanisolvens* (*P* = 0.003 and *P* < 0.001), *M. flocculare* (*P* = 0.026 and *P* = 0.011), and *L. songhuajiangensis* (*P* = 0.029 and *P* = 0.049), while ADG was positively correlated with *L. reuteri* (*P* = 0.014), *B. xylanisolvens* (*P* = 0.009), and *M. flocculare* (*P* = 0.021). In the fecal microbiota, taxa enriched in HD group also showed negative correlations with ADG, ADFI, and final body weight, though these relationships were not statistically significant (Fig. 6B). Unlike the nasal microbiota, these taxa showed a trend toward positive association with FCR.

Associations between gut microbiota-derived metabolites and growth performance showed a trend of positive correlations, although not significant (*P* > 0.05). Propionate and lactate concentrations tended to correlate with FCR, ADFI, body weight, and ADG; butyrate with FCR, ADFI, and body weight; and acetate with FCR and ADFI (Fig. 6C). In addition, a significant positive correlation was observed between lactate and TNF-α (*P* = 0.048) (Fig. 6D).

## Discussion

In this study, we assessed the impact of stocking density on pig physiology and bacterial communities. A low space allowance (HD) was implemented to stimulate crowding-induced stress, similar to a previous study [20], while a control group (CON) was maintained under established welfare standards for finishing pigs [28, 7]. This setup focused on finishing pigs, as the effects of stocking density may vary across growth stages, with younger pigs generally showing greater sensitivity due to immature immune systems and less stable bacterial communities [17].

Finishing pigs reared under high stocking density showed reduced growth performance compared to pigs in the CON group, with lower ADG and ADFI, suggesting that crowding adversely affected feed intake and efficiency. These findings align with Zhang et al. [63], who reported reduced ADG and ADFI under restricted space​ (0.38 m² per pig), potentially due to greater competition for food and chronic stress. Although nutrient digestibility did not differ significantly, apparent total tract digestibility tended to decrease under crowded conditions, indicating a possible impairment in the gut’s capacity to utilize feed efficiently [8, 63]. Together, these results indicate that providing sufficient space can support more efficient feed utilization and growth, as pigs with higher space allowances tended to gain weight faster than those in crowded conditions.

Pigs in the HD group also exhibited altered blood parameters indicative of stress, including elevated cortisol, suggesting probable chronic physiological stress that could influence immune and metabolic functions [27]. A similar increase in blood cortisol was described in growing pigs at 0.48–0.38 m² per pig compared to those at 0.64 m² per pig​ [63]. These findings point out that high stocking density may act as a chronic stressor, potentially affecting physiological outcomes.

The coughing index, used as an indicator of respiratory distress, was higher in pigs under high stocking density, likely reflecting deteriorated air quality and increased pathogen exposure when animals are packed more densely [37, 58]. Elevated ammonia and other environmental stressors may have contributed to changes in the nasal microbiota and coughing, as higher ammonia levels have been associated with reductions in beneficial taxa such as *Lactobacillus* and increases in opportunistic pathogens like *Moraxella* and *Streptococcus* ​ [54]. In contrast, pigs in the CON group, housed at a lower stocking density, likely experienced better air quality, as observed with their lower coughing index.

Nasal and gut microbiota play important functions in immunity, metabolism, and disease resistance in pigs [23, 30, 40]. Behavioral changes associated with high stocking density, such as altered social interactions and feeding patterns, can drive shifts in both nasal and gut microbial communities [34]. In this study, high stocking density resulted in significant reduction in Shannon diversity in the nasal microbiota, but not in the fecal microbiota, suggesting that the nasal community may be more sensitive to environmental stressors than the gut microbiota [14, 50, 64]. Beta-diversity analyses further revealed statistically significant, though modest, differences between the CON and HD groups in both nasal and fecal bacterial communities, indicating measurable yet limited shifts in microbial community composition under high stocking density. Similar patterns of disruption have been documented in pigs experiencing respiratory infections or antibiotic treatment. Zeineldin et al. [60] showed that antibiotics reduce both diversity and richness in the nasal microbiota, while pigs affected by Glässer’s disease exhibit lower nasal microbial diversity compared to healthy pigs [11]. Although the magnitude of change observed in our study is smaller, these parallels suggest that high stocking density can still contribute to detectable bacterial community disturbances reminiscent of those observed under antibiotic exposure or respiratory disease.

While full-length 16S rRNA sequencing of the V1-V9 regions was employed in this study to increase taxonomic resolution, the overall patterns of microbial shifts are generally consistent with previous studies using partial 16S rRNA sequencing in pig gut under high stocking density, although some differences were also observed in diversity metrics. One study using the V3-V4 regions also observed a non-significant decrease in alpha-diversity indices in the fecal microbial community of growing pigs reared at 0.55 m² per pig [20]. Similarly, another study examining the jejunum, ileum, and cecum microbiota of growing pigs using the V4 region also reported non-significant reductions in bacterial richness, particularly in the jejunum, under high stocking density (0.82 m² per pig) compared to medium (1.23 m² per pig) and low (2.46 m² per pig) stocking densities [25]. However, unlike these studies, we observed significant differences in beta-diversity, which may reflect differences in sample size, growth stage, or sequencing method. This highlights the significance of the present study in advancing the understanding of how stocking density affects gut microbial diversity in pigs, and in presenting new perspectives on its impact on the largely unexplored nasal microbiota.

At the taxonomic level, the nasal microbiota was primarily dominated by Proteobacteria and Firmicutes, with *Moraxella* as the most abundant genus, consistent with prior reports [29, 40, 46]. High stocking density resulted in the enrichment of *R*. *nasimurium*, *P. faecalis*, and *Globicatella* sp., alongside a reduction in several *Lactobacillus* species. *Rothia*, including *R. nasimurium*, is a common nasal colonizer in pigs [39]. Although its clinical relevance in pigs remains unclear, its enrichment is noteworthy, as *R. nasimurium* has been reported as a potential opportunistic and multidrug-resistant pathogen in poultry, particularly linked to feather loss and disease outbreaks [61]. Similarly, *P*. *faecalis* and *Globicatella* sp. were detected only in the HD group, indicating possible crowding-associated microbial changes. *Psychrobacter* species, including *P. faecalis*, are opportunistic pathogens known for nosocomial and environmental transmission in other hosts [53], while *Globicatella* has limited taxonomic characterization but includes species such as *G. sanguinis* implicated in opportunistic human infections [32]. The enrichment of these taxa under crowded conditions likely mirrors ecological shifts or dysbiosis rather than direct pathogenicity, highlighting the need for further research to clarify their potential impact on respiratory health in pigs.

In the fecal microbiota of finishing pigs, Firmicutes was the dominant phylum, followed by Bacteroidota, consistent with previous studies on the same breed of pigs [35, 51]. High stocking density was associated with a significant reduction in *Lactobacillus* and an increase in *Clostridium sensu stricto 1* and *Terrisporobacter*. These changes, observed at both genus and species levels, resemble patterns reported in the gut microbiota of chickens, where a decrease in *Lactobacillus* preceded overgrowth of *Clostridium sensu stricto 1*, a contributor to necrotic enteritis [59]. *Terrisporobacter*, although less studied, has been implicated in rare but severe human infections [49], suggesting that its increased abundance under crowded conditions may carry potential health implications.

Across both nasal and fecal bacterial communities, high stocking density was associated with a reduction in beneficial *Lactobacillus* species, including *L*. *reuteri*, *L*. *songhuajiangensis*, *L*. *heilongjiangensis*, *L. amylovorus*, and *L. johnsonii*. In the nasal microbiota, *L*. *reuteri* and *L*. *songhuajiangensis* were positively associated with body weight, ADG, and ADFI, suggesting potential links to growth performance. Previous studies have reported the persistence of *Lactobacillus* in the nasal cavity of pigs, where it contributes to respiratory health and immune regulation [11, 29, 40, 46]. In the gut, the reduction in *Lactobacillus* coincided with a significant decrease in fecal lactate concentration under high stocking density, indicating possible disruption of lactate-dependent metabolic pathways. Lactate produced by *Lactobacillus* helps maintain a low-pH environment that suppresses pathogenic bacteria [52, 57, 62] and serves as an intermediate in cross-feeding interactions that generate SCFA such as acetate, propionate, and butyrate, which are essential for energy metabolism, immune function, and gut integrity [2, 52]. Moreover, *Lactobacillus* has been shown to support stress adaptation and recovery in pigs [6] and promote host growth and stress resilience [3]. In the current study, we also observed a positive correlation between lactate and TNF-α, which may suggest a possible immunomodulatory role of lactate [66]. Lactate, produced by both host and microbial cells, has been implicated in immune regulation and inflammation by promoting TNF-α production through glycolysis-dependent differentiation of CD8^+^ T cells into effector cells and by enhancing M1 macrophage activity [13]. Although overall microbiota-derived lactate levels were reduced under high stocking density and TNF-α remained unchanged, the positive correlation between the two suggests that reduced lactate may reflect microbial or metabolic disruption under stress, potentially influencing inflammatory responses in pigs. Taken together, the observed reductions in *Lactobacillus* abundance in both nasal and fecal bacterial communities, along with decreased fecal lactate levels, indicate that high stocking density may shift the microbial ecosystem toward opportunistic and potentially pathogenic bacteria while depleting taxa associated with growth, stress resilience, and respiratory and gut health.

Functional predictions indicated alterations in microbial metabolic pathways, with potential implications for energy metabolism, carbohydrate utilization, and stress responses. While these results are suggestive, functional inferences based on 16S rRNA sequencing remain tentative, and further metagenomic or metabolomic studies are warranted.

This study has some limitations that should be considered. First, this study focused on the effects of stocking density in finishing pigs over a 4-week period. Longitudinal sampling would help track whether the observed physiological and microbial changes are sustained, reversible, or progressive over longer durations. Moreover, because this study was conducted in finishing pigs, it remains uncertain whether similar responses would occur in younger pigs, such as during weaning or growing, when animals may be more sensitive to environmental stressors. Second, environmental parameters such as ammonia concentration, dust levels, and ventilation were not measured but may warrant inclusion in future research to better disentangle their potential impact on respiratory health and the nasal microbiota. Although the coughing index served as a general indicator of respiratory distress, additional clinical and behavioral assessments could complement the present findings. Third, this study did not measure fecal glucocorticoid metabolites to assess chronic stress. Incorporating such non-invasive measures could supplement serum cortisol analysis. Finally, pigs were randomly allocated into groups to minimize initial stress differences, allowing for meaningful post-treatment comparisons. Including baseline measurements in future studies could enable within-subject stress tracking over time and strengthen causal interpretations.

## Conclusions

This study revealed that high stocking density negatively affected growth performance in finishing pigs, as evidenced by reduced final body weight, ADG, and ADFI, alongside elevated cortisol levels and stress-induced coughing. Microbiota analyses showed decreased microbial diversity in both the nasal and fecal microbiota, with a more significant decline in the nasal bacterial community of the HD group. High stocking density increased the abundance of *R*. *nasimurium*, *P*. *faecalis*, and *Globicatella* sp. in the nasal microbiota, while in the fecal microbiota it promoted the proliferation of *Clostridium sensu stricto* 1 and *Terrisporobacter* and reduced *Lactobacillus*, particularly *L*. *amylovorus* and *L*. *johnsonii*. These compositional changes coincided with decreased fecal lactate concentrations, suggesting potential disruptions in gut metabolic function. Collectively, these findings indicate that high stocking density may alter microbial community composition, potentially contributing to reduced growth performance and heightened stress responses. Even modest microbial shifts observed in crowded pens were associated with lower growth and increased stress, highlighting the importance of providing adequate space to maintain productivity and respiratory health. Overall, this study offers new insights into how crowding influences microbial dynamics and health in finishing pigs.

## Supplementary Information

Below is the link to the electronic supplementary material.

Supplementary Material 1

## Data Availability

All standard sequence format (.fastq) files generated by PacBio Sequel containing all raw sequence reads have been deposited at the National Center for Biotechnology (NCBI) Sequence Read Archive (SRA) repository (BioProject accession number: PRJNA1205809; https:/www.ncbi.nlm.nih.gov/sra/PRJNA1205809).

## Abbreviations

*k*: Space allowance coefficient
QIIME 2: Quantitative insights into microbial ecology 2
PCoA: Principal coordinate analysis
LEfSe: Linear discriminant analysis Effect Size
HPLC: High-performance liquid chromatography
SCFA: Short-chain fatty acids
PERMANOVA: Permutational multivariate analysis of variance
LDA: Linear discriminant analysis
IL-6: Interleukin-6
TNF-α: Tumor necrosis factor-alpha
FCR: Feed conversion ratio
ADFI: Average daily feed intake
ADG: Average daily gain
