# Supplementary material for: High stocking density triggers stress-induced physiological changes and alters nasal and fecal microbiota in finishing pigs
Source: Porcine Health Manag. 2026 Apr 24;12:33. doi: 10.1186/s40813-026-00515-3 (PMC13262304; doi:10.1186/s40813-026-00515-3)
Supplement: Supplementary file 1 — Supplementary Material 1 [file 40813_2026_515_MOESM1_ESM.pdf]

High stocking density triggers stress-induced physiological changes and alters nasal and fecal microbiota in finishing pigs

Robie Vasquez [§,†], Van Aldren Cañas[§,†], Sungbo Cho[§,#], Ji Hoon Song[§], Jae Seung Lee[§], Chaibin Lim[§,#], Dae-Kyung Kang[§,#,*], In Ho Kim[§,#,*]

Supplementary Materials

Supplementary Table S1. Composition of finishing pig diet (as fed-basis).

Item	Basal diet
**Ingredients (%)**	
Corn	83.46
Soybean meal	11.70
Tallow	0.17
Molasses	2.00
MCP	0.80
Limestone	0.66
Lysine (78%)	0.36
Methionine (98%)	0.02
Threonine (98%)	0.11
Tryptophan (98%)	0.02
Salt	0.20
Mineral mix[1]	0.20
Vitamin mix[2]	0.20
Choline (25%)	0.10
Total	100.00
**Calculated value**	
Crude protein, %	13.00
Metabolizable Energy, kcal/kg	3300
Calcium, %	0.52
Phosphorus, %	0.47
Lysine, %	0.84
Methionine, %	0.25
Threonine, %	0.56
Tryptophan, %	0.15
Crude Fat, %	3.25
Crude Fiber, %	2.11
Crude Ash, %	3.40

[1] Provided per kg diet: Fe, 100 mg as ferrous sulfate; Cu, 17 mg as copper sulfate; Mn, 17 mg as manganese oxide; I, 0.5 mg as potassium iodide; and Se, 0.3 mg as sodium selenite.

[2] Provided per kilograms of diet: vitamin A, 10,800 IU; vitamin D3, 4,000 IU; vitamin E, 40 IU; vitamin K3, 4 mg; vitamin B1, 6 mg; vitamin B2, 12 mg; vitamin B6, 6 mg; vitamin B12, 0.05 mg; biotin, 0.2 mg; folic acid, 2 mg; niacin, 50 mg; D-calcium pantothenate, 25 mg.

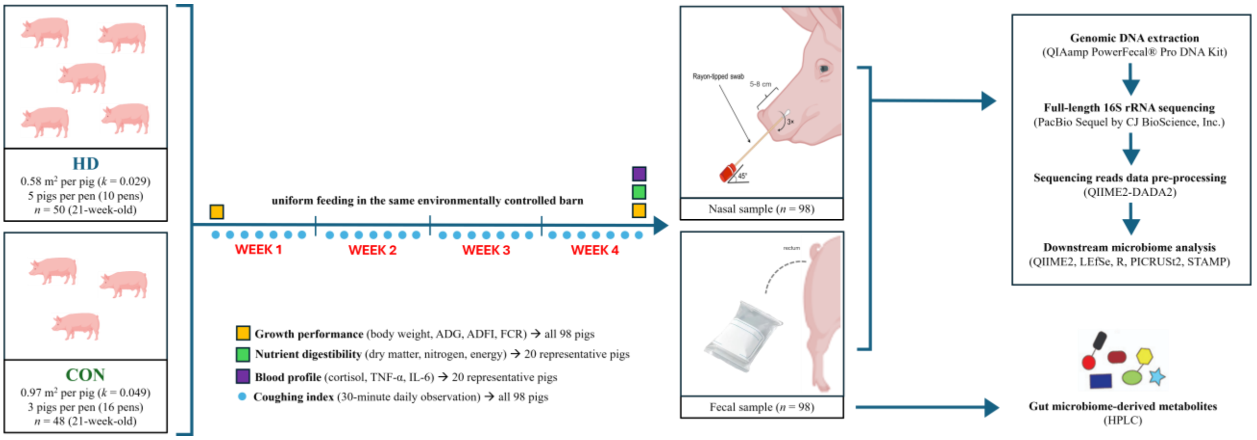

Supplementary Fig. S1. Experimental setup and sampling scheme of the stocking density experiment in finishing pigs.

Supplementary Table S2. Next-generation sequencing of nasal and fecal samples from finishing pigs.

Groups	Total raw reads	Valid reads[1]					
		Total	Minimum	Maximum	Average	Good's coverage (%)	Average length (bp)
**Nasal**							
CON	990,375	409,517	2,208	14,760	8,531.60	100	1,444.39
HD	1,091,826	404,358	3,287	14,123	8,087.16	100	
**Fecal**							
CON	487,006	54,860	397	2,700	1,142.92	100	1,458.90
HD	491,206	51,846	254	2,226	1,036.92	100	

Abbreviations: CON = control (*n* = 48); HD = high stocking density (*n* = 50).

[1] Data were pre-processed using DADA2 plugin in QIIME 2.

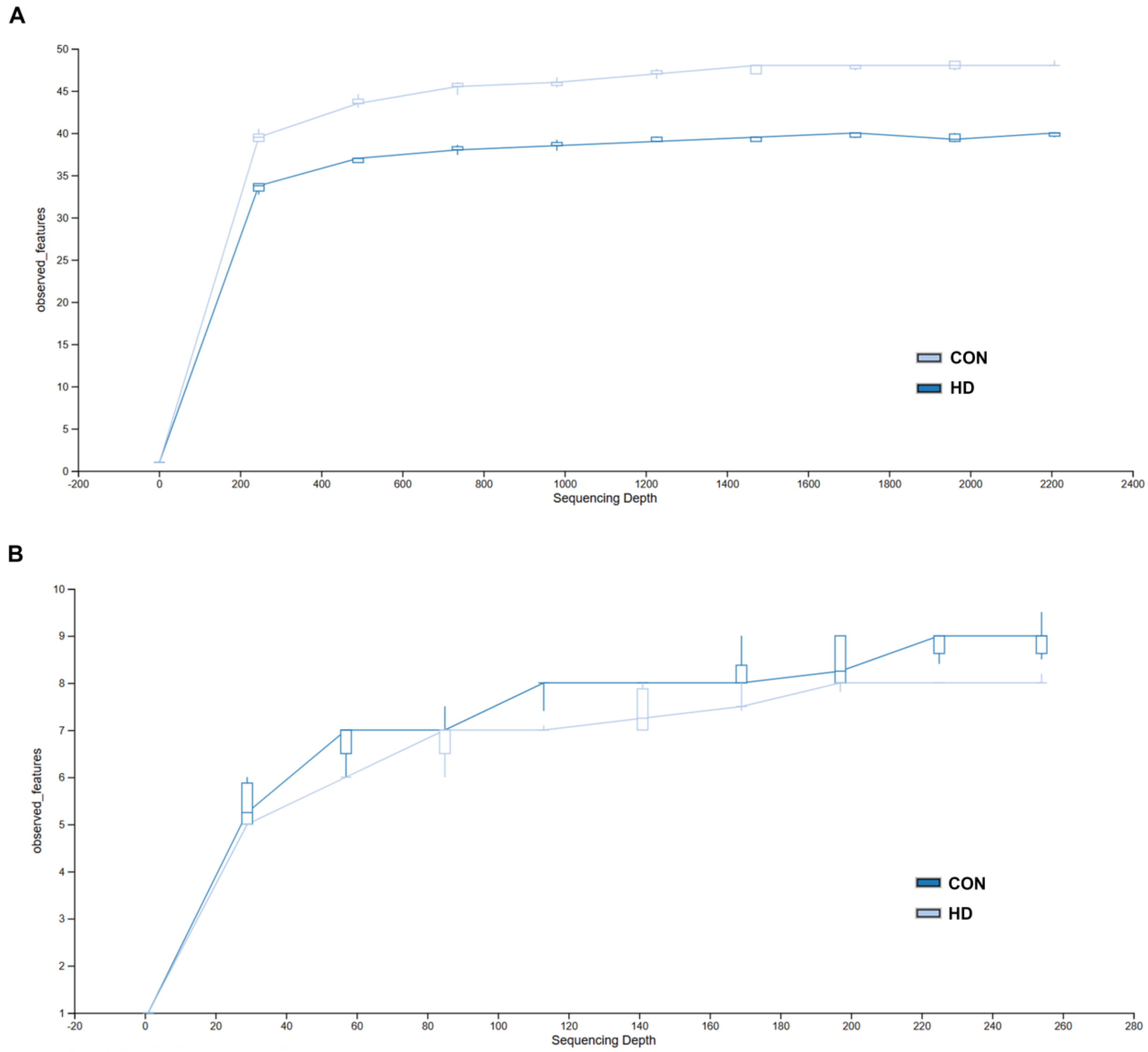

Supplementary Fig. S2. Alpha-rarefaction curves for observed features in nasal (A) and fecal (B) samples of finishing pigs. Sequencing depths were at 2,208 and 254, respectively. Abbreviations: CON = control (*n* = 48); HD = high stocking density (*n* = 50).

Supplementary Table S3. Effect of stocking density on nasal and fecal microbiota alpha-diversity in finishing pigs.

Alpha-diversity indices	Mean[1]		P-value[2]
	CON	HD	
**Nasal**			
Shannon Entropy	4.56 ± 0.081	4.34 ± 0.061	0.009*
Simpson	0.92 ± 0.007	0.92 ± 0.004	0.060
**Fecal**			
Shannon Entropy	2.05 ± 0.083	1.93 ± 0.097	0.325
Simpson	0.64 ± 0.021	0.61 ± 0.025	0.611

Abbreviations: CON = control (n = 48); HD = high stocking density (n = 50).

[1] Mean ± SEM (standard error of mean)
[2] The *P*-values were determined using Mann-Whitney U test ($P < 0.05$).
* Statistically significant *P*-value.

Supplementary Table S4. Relative abundance (%) at phylum, genus and species levels in nasal samples of finishing pigs.

Taxa	Mean[1]		P-value[2]
	CON	HD	
**Phylum**			
Proteobacteria	50.77 ± 2.058	50.12 ± 1.602	0.642
Firmicutes	30.66 ± 1.940	29.56 ± 2.135	0.248
Bacteroidota	12.89 ± 1.379	10.26 ± 1.266	0.148
Actinobacteriota	5.50 ± 0.758	9.96 ± 1.112	0.005*
Patescibacteria	0.09 ± 0.024	0.09 ± 0.030	0.604
Fusobacteriota	0.09 ± 0.082	0.005 ± 0.005	0.528
**Genus**			
Moraxella	36.44 ± 2.333	38.14 ± 1.947	0.534
Streptococcus	12.57 ± 0.840	11.40 ± 1.208	0.073
Rothia	5.50 ± 0.758	9.94 ± 1.109	0.005*
Bergeyella	6.68 ± 1.127	6.58 ± 1.099	0.823
Actinobacillus	9.09 ± 1.413	4.26 ± 0.980	0.001*
Companilactobacillus	4.37 ± 1.243	6.20 ± 1.391	0.332
Filobacterium	5.16 ± 0.949	2.33 ± 0.693	0.001*
Carnobacteriaceae unassigned	3.61 ± 0.621	3.40 ± 0.597	0.703
Neisseriaceae unassigned	2.69 ± 0.417	2.71 ± 0.485	0.974
Mannheimia	1.98 ± 0.547	3.29 ± 1.003	0.208
Levilactobacillus	1.53 ± 0.487	2.04 ± 0.569	0.709
Lactobacillus	1.22 ± 0.234	1.89 ± 0.862	0.057
Clostridium sensu stricto 1	1.63 ± 0.276	1.23 ± 0.270	0.267
Chryseobacterium	0.69 ± 0.201	1.33 ± 0.302	0.062
Terrisporobacter	0.82 ± 0.141	0.65 ± 0.132	0.351
Limosilactobacillus	0.69 ± 0.183	0.67 ± 0.213	0.311
Lactiplantibacillus	0.80 ± 0.196	0.55 ± 0.154	0.222
Mycoplasma	1.21 ± 0.325	0.17 ± 0.071	<0.001*
Weissella	0.88 ± 0.448	0.15 ± 0.080	0.326
Neisseria	0.43 ± 0.169	0.43 ± 0.179	0.916
Acinetobacter	0.13 ± 0.103	0.62 ± 0.543	0.689
Romboutsia	0.37 ± 0.092	0.21 ± 0.098	0.067
Psychrobacter	0	0.55 ± 0.312	0.026*
Globicatella	0.13 ± 0.032	0.36 ± 0.087	0.053
Other genera	1.38 ± 0.531	0.90 ± 0.221	0.513
**Species**			
Moraxella sp.	16.02 ± 1.757	17.96 ± 1.620	0.240
Moraxella unassigned	11.51 ± 1.646	12.09 ± 1.083	0.288
Streptococcus suis	9.60 ± 0.871	8.64 ± 0.985	0.137
Rothia nasimurium	5.48 ± 0.759	9.94 ± 1.109	0.005*

Moraxella pluranimalium	4.89 ± 0.911	6.77 ± 1.138	0.135
Glaesserella parasuis	6.85 ± 1.083	2.91 ± 0.564	0.002*
Lactobacillus paralimentarius	3.53 ± 1.025	5.47 ± 1.287	0.356
Filobacterium uncultured	5.16 ± 0.949	2.33 ± 0.693	0.001*
Carnobacteriaceae unassigned	3.61 ± 0.621	3.40 ± 0.597	0.703
Bergeyella zoohelcum	4.25 ± 1.130	2.49 ± 0.791	0.138
Bergeyella porcorum	2.43 ± 0.461	4.08 ± 0.925	0.123
Neisseriaceae unassigned	2.69 ± 0.417	2.71 ± 0.485	0.974
Mannheimia sp.	1.98 ± 0.548	3.29 ± 1.003	0.180
Moraxella boevrei	4.02 ± 1.058	1.32 ± 0.362	0.007*
Actinobacillus unassigned	2.22 ± 0.599	1.35 ± 0.481	0.010*
Streptococcus pluranimalium	2.28 ± 0.520	1.06 ± 0.279	0.024*
Lactobacillus amylovorus	1.06 ± 0.192	1.86 ± 0.862	0.063
Clostridium sensu stricto 1 uncultured	1.46 ± 0.237	1.15 ± 0.253	0.220
Streptococcus acidominimus	0.67 ± 0.243	1.67 ± 0.816	0.427
Lactobacillus namurensis	0.87 ± 0.400	1.35 ± 0.471	0.302
Chryseobacterium unassigned	0.69 ± 0.201	1.33 ± 0.302	0.062
Terrisporobacter uncultured	0.82 ± 0.141	0.65 ± 0.200	0.351
Lactiplantibacillus unassigned	0.79 ± 0.185	0.55 ± 0.154	0.222
Companilactobacillus unassigned	0.64 ± 0.255	0.65 ± 0.200	0.571
Mycoplasma hyorhinis	0.95 ± 0.315	0.15 ± 0.071	0.001*
Psychrobacter faecalis	0	0.51 ± 0.285	0.026*
Mesomycoplasma flocculare	0.26 ± 0.118	0.02 ± 0.013	0.017*
Lactobacillus songhuajiangensis	0.11 ± 0.048	0	0.001*
Lactobacillus heilongjiangensis	0.02 ± 0.012	0	0.039*
Bacteroides xylanisolvens	0.05 ± 0.019	0	0.001*
Other species	5.10 ± 0.898	4.30 ± 0.824	0.752

Abbreviations: CON = control (n = 48); HD = high stocking density (n = 50).

[1] Mean ± SEM (standard error of mean)
[2] The *P*-values were determined using Mann-Whitney U test ($P < 0.05$).
* Statistically significant *P*-value.

Supplementary Table S5. Relative abundance (%) at phylum, genus and species levels in fecal samples of finishing pigs.

Taxa	Mean[1]		*P*-value[2]
	CON	HD	
**Phylum**			
Firmicutes	94.66 ± 1.420	94.87 ± 1.328	0.905
Bacteroidota	3.94 ± 1.229	3.86 ± 1.085	0.936
Proteobacteria	0.77 ± 0.632	0.51 ± 0.401	0.467
Actinobacteriota	0.14 ± 0.094	0.32 ± 0.169	0.708
Desulfobacterota	0.17 ± 0.174	0.25 ± 0.231	0.592
Planctomycetota	0.26 ± 0.139	0.14 ± 0.067	0.722
Spirochaetota	0.06 ± 0.042	0.02 ± 0.010	0.730
Verrucomicrobiota	0	0.02 ± 0.018	0.337
**Genus**			
Lactobacillus	45.35 ± 3.801	30.84 ± 4.336	0.019*
Clostridium sensu stricto 1	24.08 ± 2.773	34.33 ± 3.277	0.026*
Terrisporobacter	9.13 ± 1.246	15.69 ± 1.808	0.010*
Limosilactobacillus	6.17 ± 1.613	4.51 ± 1.044	0.628
Rikenellaceae RC9 gut group	2.64 ± 0.936	1.31 ± 0.386	0.867
Romboutsia	1.56 ± 0.567	2.17 ± 0.809	1.000
Shuttleworthia	0.68 ± 0.308	2.38 ± 1.499	0.559
Dialister	1.70 ± 0.572	1.05 ± 0.387	0.480
Bacteroidales p2534 18B5 gut group	0.41 ± 0.296	1.49 ± 0.812	0.208
Turicibacter	0.70 ± 0.198	0.76 ± 0.154	0.377
Escherichia-Shigella	0.77 ± 0.632	0.51 ± 0.401	0.467
[Eubacterium] coprostanoligenes group	0.93 ± 0.769	0.21 ± 0.195	0.369
Sarcina	1.04 ± 0.578	0	0.020*
Succiniclasticum	0.92 ± 0.442	0.04 ± 0.041	0.042*
Prevotella 7	0.27 ± 0.180	0.63 ± 0.293	0.728
Streptococcus	0.44 ± 0.332	0.33 ± 0.166	0.530
Ruminococcus	0.17 ± 0.099	0.48 ± 0.217	0.367
Catenibacterium	0.19 ± 0.085	0.44 ± 0.170	0.283
Prevotella 9	0.33 ± 0.192	0.14 ± 0.088	0.905
Olsenella	0.14 ± 0.094	0.29 ± 0.157	0.708
Desulfovibrio	0.17 ± 0.174	0.25 ± 0.231	0.592
Pirellulaceae p1088 a5 gut group	0.26 ± 0.139	0.14 ± 0.067	0.722
Pseudoramibacter	0.23 ± 0.137	0.11 ± 0.080	0.608
Syntrophococcus	0.31 ± 0.210	0	0.076
Other genera	1.41 ± 0.330	1.88 ± 0.464	0.238
**Species**			
Lactobacillus amylovorus	42.19 ± 4.023	28.21 ± 4.333	0.021*
Clostridium sensu stricto 1 uncultured	24.07 ± 2.773	34.12 ± 3.258	0.028*

Terrisporobacter uncultured	9.13 ± 1.246	15.69 ± 1.808	0.010*
Lactobacillus.reuteri	5.80 ± 1.559	4.51 ± 1.044	0.683
Lactobacillus.johnsonii	3.16 ± 1.537	2.64 ± 1.725	0.035*
Rikenellaceae RC9 gut group uncultured	2.64 ± 0.936	1.31 ± 0.386	0.867
Romboutsia unassigned	1.56 ± 0.567	2.17 ± 0.809	1.000
Bacteroides xylanisolvens	1.70 ± 0.572	1.05 ± 0.387	0.480
Shuttleworthia uncultured	0.50 ± 0.289	1.92 ± 1.493	0.989
Bacteroidales p2534 18B5 gut group uncultured	0.41 ± 0.296	1.49 ± 0.812	0.208
Turicibacter sp.	0.70 ± 0.198	0.76 ± 0.154	0.377
Escherichia-Shigella unassigned	0.77 ± 0.632	0.51 ± 0.401	0.467
[Eubacterium] coprostanoligenes group uncultured	0.90 ± 0.769	0.20 ± 0.195	0.300
Sarcina unassigned	1.04 ± 0.578	0	0.020*
Succiniclasticum uncultured	0.92 ± 0.442	0.04 ± 0.041	0.042*
Prevotella 7 uncultured rumen	0.27 ± 0.180	0.63 ± 0.293	0.728
Catenibacterium mitsuokai	0.19 ± 0.085	0.44 ± 0.170	0.283
Desulfovibrio metagenome	0.17 ± 0.174	0.25 ± 0.231	0.592
Pirellulaceae p1088 a5 gut group uncultured	0.26 ± 0.139	0.14 ± 0.67	0.722
Shuttleworthia uncultured rumen	0.16 ± 0.124	0.22 ± 0.109	0.436
Lactobacillus mucosae	0.37 ± 0.367	0	0.317
Ruminococcus unassigned	0.15 ± 0.098	0.19 ± 0.135	0.768
Streptococcus alactolyticus	0.01 ± 0.012	0.32 ± 0.166	0.177
Prevotella 9 unassigned	0.25 ± 0.177	0.08 ± 0.064	0.942
Other species	2.66 ± 0.678	3.09 ± 0.688	0.340

Abbreviations: CON = control (n = 48); HD = high stocking density (n = 50).

[1] Mean ± SEM (standard error of mean)
[2] The *P*-values were determined using Mann-Whitney U test ($P < 0.05$).
* Statistically significant *P*-value.

Supplementary Table S6. Effect of stocking density on pig gut microbiota-derived lactate and SCFAs concentrations.

Metabolites	Mean[1]		P-value[2]
	CON	HD	
Lactate	16.93 ± 3.159	12.37 ± 3.538	0.005*
Acetate	199.80 ± 8.464	189.88 ± 5.658	0.548
Propionate	125.25 ± 5.616	115.89 ± 4.545	0.207
Butyrate	60.82 ± 3.333	57.06 ± 2.858	0.596

Abbreviations: CON = control (n = 48); HD = high stocking density (n = 50).

[1] Mean ± SEM (standard error of mean)
[2] The P-values were determined using Mann-Whitney U test ($P < 0.05$).
* Statistically significant P-value.
